# High-resolution MRI assessment of dactylitis in psoriatic arthritis shows flexor tendon pulley and sheath-related enthesitis

**DOI:** 10.1136/annrheumdis-2014-205839

**Published:** 2014-09-26

**Authors:** Ai Lyn Tan, Eiji Fukuba, Nicola Ann Halliday, Steven F Tanner, Paul Emery, Dennis McGonagle

**Affiliations:** 1 NIHR Leeds Musculoskeletal Biomedical Research Unit, Chapel Allerton Hospital, Leeds, UK; 2 Leeds Institute of Rheumatic and Musculoskeletal Medicine, University of Leeds, Leeds, UK; 3 Faculty of Medicine, Department of Radiology, Shimane University, Japan; 4 Department of Medical Physics and Engineering, Leeds Teaching Hospitals NHS Trust, Leeds, UK

## Abstract

**Objective:**

Dactylitis is a hallmark of psoriatic arthritis (PsA) where flexor tenosynovitis is common. This study explored the microanatomical basis of dactylitis using high-resolution MRI (hrMRI) to visualise the small entheses around the digits.

**Methods:**

Twelve patients with psoriatic dactylitis (4 fingers, 8 toes), and 10 healthy volunteers (6 fingers, 4 toes) had hrMRI of the digits using a ‘microscopy’ coil and contrast enhancement. All structures were evaluated including the tendons and ligaments, related enthesis organs, pulleys, volar/plantar plates and tendon sheaths.

**Results:**

In dactylitis, collateral ligament enthesitis was seen in nine digits (75%), extensor tendon enthesitis in six digits (50%), functional enthesitis (5 digits, 42%), abnormal enhancement at the volar plates (2/5 joints, 40%) and the plantar plate (1/5 joints, 20%). Nine cases (75%) demonstrated flexor tenosynovitis, with flexor tendon pulley/flexor sheath microenthesopathy observed in 50% of all cases. Less abnormalities which were milder was observed in the normal controls, none of whom had any signal changes in the tendon pulleys or fibrous sheaths.

**Conclusions:**

This study provides proof of concept for a link between dactylitis and ‘digital polyenthesitis’ including disease of the miniature enthesis pulleys of the flexor tendons, further affirming the concept of enthesitis in PsA.

## Introduction

Dactylitis, or sausage digits, is a hallmark of psoriatic arthritis (PsA) occurring in around 40% of cases.^1^ It has been suggested that enthesitis is the primary lesion in spondyloarthritis including PsA.^2^ However, evidence suggests that dactylitis is primarily related to flexor tenosynovitis.^3^ Using MRI, Olivieri *et al*^4^ ^5^ reported no evidence for enthesitis in dactylitis. Other imaging studies also reported soft tissue oedema, joint synovitis and osteitis but not enthesitis.^6^ ^7^

Previously, we have used high-resolution MRI (hrMRI) to explore the microanatomical basis for distal interphalangeal (IP) joint PsA where enthesitis was common.^8^ We have also noted ‘functional entheses’ disease at the extensor tendon and ligament enthesitis of the distal IP joint on cases of dactylitis on hrMRI,^9^ ^10^ where tendons and ligaments wrap around bony pulleys and are associated with fibrocartilage that reduces compression and shear.^11^ The present study used hrMRI to explore the basis for PsA dactylitis by looking at the entheses and functional entheses-related structures. Given the dactylitis link with flexor tenosynovitis, we looked at the small entheses or flexor tendon pulleys that are sites of high mechanical stress and that have thus far not been explored as potential enthesis-related drivers of dactylitis.

## Subjects and methods

### Subjects

Twelve patients (3 females, 9 males: mean age 43.9 years (range 23 years–59 years)) with PsA dactylitis (mean disease duration 56 months (range 1month–299 months)), and 10 healthy volunteers with no arthritis or dactylitis (7 females, 3 males: mean age 37.7 years (range 24 years–59 years)) were recruited, and provided written informed consent, following ethics committee approval. The PsA patients were diagnosed based on clinical finding of typical appearance of dactylitis; all except 1 patient (n=11) fulfilled CASPAR (ClASsification of Psoriatic Arthritis Criteria) criteria,^12^ the one patient who did not fulfil the criteria was negative for rheumatoid factor and positive for HLA-B27.

### Magnetic Resonance Imaging

MRI was performed using a 1.5-Tesla whole-body scanner (Philips, Gyroscan Intera), and either a 23 mm or a 47 mm diameter microscopy hrMRI surface coil to ensure adequate coverage of the region of interest for optimum resolution, with the coil being placed on the worst affected dactylitic fingers or toes. A dactylitic digit in each patient and a randomly selected normal digit for the controls were imaged. The imaging sequences are as previously reported.^13^ The following sequences were used: T2-weighted sagittal or coronal with a fat-selective presaturation pulse, water-selective excitation three-dimensional sagittal, proton-density-weighted turbo-spin-echo coronal, T1-weighted spin-echo coronal and axial, contrast-enhanced fat-saturated T1-weighted coronal, sagittal and axial. Contrast-enhanced MRI was obtained after intravenous injection of 10 mL of gadolinium diethylene-triaminepentaacetic acid.

### Image analysis

The MRIs were analysed using an image analysis software package ImageJ (National Institute of Health, Maryland, USA, http://rsb.info.nih.gov/ij/). The following structures were evaluated by consensus by a radiologist (EF) and a rheumatologist (DM) who were blinded to the diagnosis of the subjects: flexor and extensor tendons, extracapsular soft tissues, bone cortex, bone marrow, intracapsular synovium, finger pulleys, toe fibrous sheaths, enthesis organs (collateral ligaments, flexor and extensor tendons), finger volar plates and toe plantar plate ligaments. Abnormalities were identified as present or absent, and signal intensity changes were semiqualitatively classified as none, mild, moderate and severe.

Enthesitis on MRI was defined as increased signal intensity adjacent to insertions of the collateral ligaments or the flexor/extensor tendons on fat-saturated T2-weighted, water-selective excitation, or contrast-enhanced fat-saturated T1-weighted images, and/or ligament swelling that extended to the entheses with increased signal as previously described.^8^ Osteophytes at the entheses were termed enthesophytes.

### Statistical analysis

Proportions of abnormalities are presented as percentages of the total observations in each group. Fisher's exact test was used to compare the proportion of abnormalities between the groups using the Statistical Package for the Social Sciences V.21.0. This is a small observational descriptive study, so the results of significance tests are presented as a guideline only.

## Results

MRI of 4 dactylitic fingers and 8 dactylitic toes in 12 PsA patients, 6 normal fingers and 4 normal toes in 10 healthy volunteers were acquired. A total of 22 dactylitic joints (5 finger joints in 4 fingers (2 IP joints of the thumbs, 2 proximal IP (PIP) joints and 1 metacarpophalangeal joint) and 17 toe joints in 8 toes (3 IP joints of the big toe, 4 distal IP (DIP) joints, 5 PIP joints and 5 metatarsophalangeal (MTP) joints)) were covered by the MRI acquisition. The normal control group consisted of 13 joints (6 finger joints in 6 fingers (1 DIP, 5 PIP joints) and 7 toe joints in 4 toes (3 DIP, 4 PIP joints)).

### Entheseal changes

**Figure 1:**
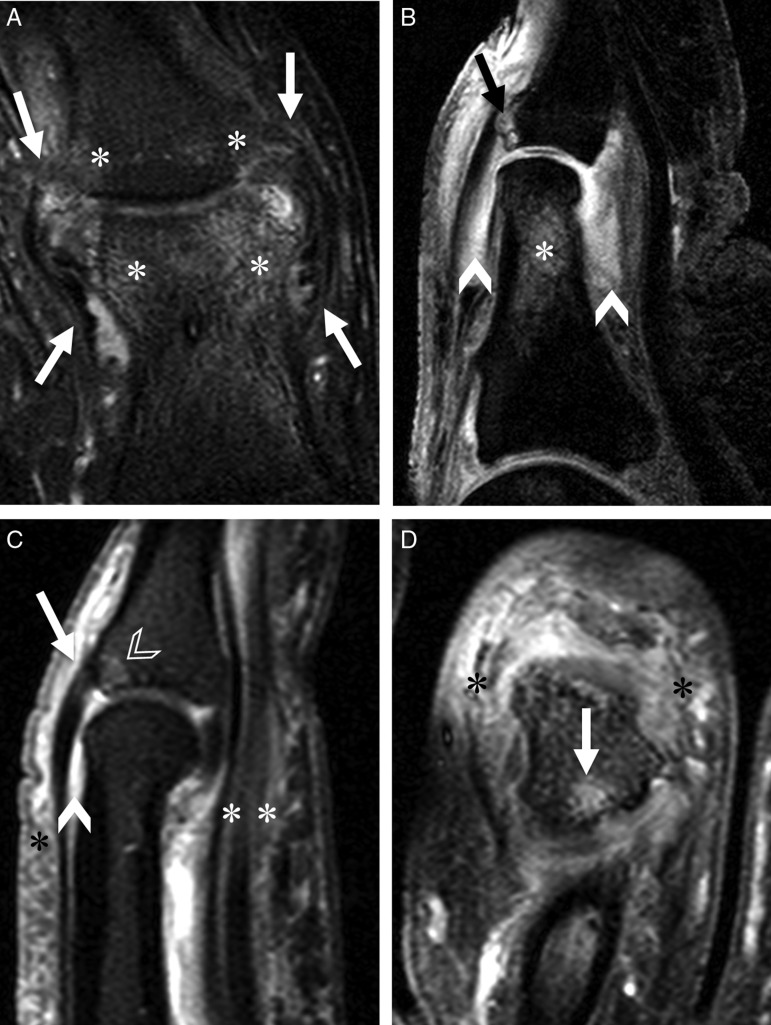
Enthesitis and osteitis in dactylitis. Fat-saturated contrast-enhanced T1-weighted images. (A) Coronal image of the left big toe of a 37-year-old man. Bilateral collateral ligament insertions and origins show mild to moderate enhancement (arrows). Mild to moderate bone marrow enhancement adjacent to the origins and insertions of the ligaments can also be seen (asterisks). (B) Sagittal image of the left big toe of same patient as (A) showing focal bone marrow enhancement at the extensor tendon insertion (arrow) and diffuse enhanced bone marrow (asterisk). Intracapsular synovium of the interphalangeal joint was swollen and moderately enhanced (arrowheads). (C) Sagittal image of the right middle finger of a 41-year-old man. The extensor tendon insertion demonstrated mild enhancement (arrow) with associated bone oedema (white outlined arrowhead). Diffuse enhancement of the flexor tenosynovium (white asterisks), extracapsular soft tissues (black asterisk), and moderate enhanced intracapsular synovium (arrowhead) in the proximal interphalangeal joint were also seen. (D) Coronal image of the right 2nd toe of a 42-year-old woman. The base of the middle phalangeal bone was partially enhanced (arrow), adjacent to the extensor tendon insertion. Diffuse soft tissue enhancement was also shown (black asterisks).

In dactylitis, enthesitis at the collateral ligament insertions were observed in 2 fingers and 7 toes (75%) (figure 1A), and of the extensor tendon insertions in 2 fingers and 4 toes (50%) (figure 1B, C). Of the 3 dactylitic digits with no ligament enthesitis, two patients had intramuscular corticosteroid 3 and 6 weeks, respectively, before the scan, both of whom also had no discernable extensor tendon enthesitis. Increased enhancement in the volar plate was seen in 2 fingers (2/5 the finger joints (40%), mild) and in the plantar plate ligament of 1 toe (1/5 MTP joint (20%), moderate). No patients showed enthesitis at the flexor tendon insertions. In the normal cohort, apart from 2 toes that showed changes of enthesitis of the extensor tendon insertions (p=0.204), there was no other evidence of enthesitis elsewhere.

### Extracapsular and synovial changes

**Figure 2:**
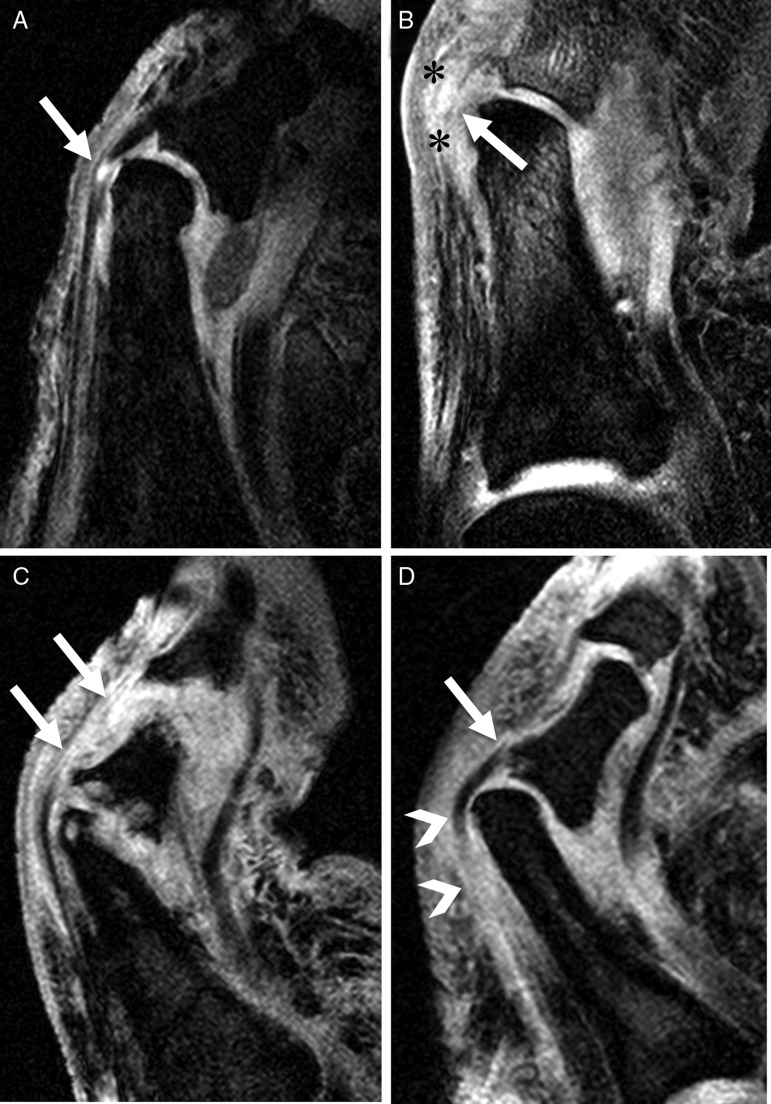
Functional enthesitis at the extensor tendons of the toes. Water-selective excitation sagittal images. (A) Focal increased signal intensity lesion in the extensor tendon of the right 2nd toe (arrow) of a 59-year-old woman. Note that this lesion was adjacent to the proximal phalangeal bone protuberance which forms a ‘functional enthesis’. (B) The left big toe of a 36-year-old man. The signal of the extensor tendon of the toe was increased diffusely at the ‘functional’ enthesis (arrow). Note that peritendinous soft tissue oedema was also shown (asterisks). (C) The right 2nd toe of a 42-year-old woman. Diffuse increased signal intensity lesion in the extensor tendon could be seen (arrows). (D) The left 4th toe of a 40-year-old man. The signal of the extensor tendon was increased diffusely near the proximal phalangeal bone protuberance (arrowheads). Enthesitis at insertion of the extensor tendon was also visible (arrow).

Eleven patients (92%) showed diffuse extracapsular soft tissue oedema (10 cases, moderate; 1 case, mild). Increased signal intensities in 5 extensor tendons (all mild) of 5 subjects (1 finger and 4 toes, 42% of all cases) were noted. The extensor tendon lesions of the dactylitic finger and 2 of the 4 toes were adjacent to the phalangeal bone protuberances (figure 2). Five joints of 4 fingers and 10 joints of 7 toes (68% of all 22 joints) exhibited synovitis. Of these, 2 joints of 2 fingers and 9 joints of 7 toes showed moderate synovitis and 3 joints of 2 fingers and one joint of one toe demonstrated mild synovitis. In the normal controls, 3 had mild, and 2 moderate appearances of extracapsular soft tissue oedema (50%, p=0.056); 5 had appearances resembling mild intracapsular enhancement (50%, p=0.056); and all extensor tendons were normal.

### Bone or bone marrow changes

In 3 fingers and 7 toes of the dactylitic cohort (83%), diffuse or focal increased bone marrow signal were observed (figure 1). One finger and 6 toes had bone erosions at insertions of the collateral ligaments. In the base of the distal phalanges of two big toes (12% of all 17 joints of the toes), enthesophytes were seen. Only 1 of the normal controls had mild bone marrow oedema in the toe (10%, p=0.002), and no other bone changes were observed in any of the normal controls.

### Flexor tendons and tendon pulley/fibrous sheath disease

**Figure 3:**
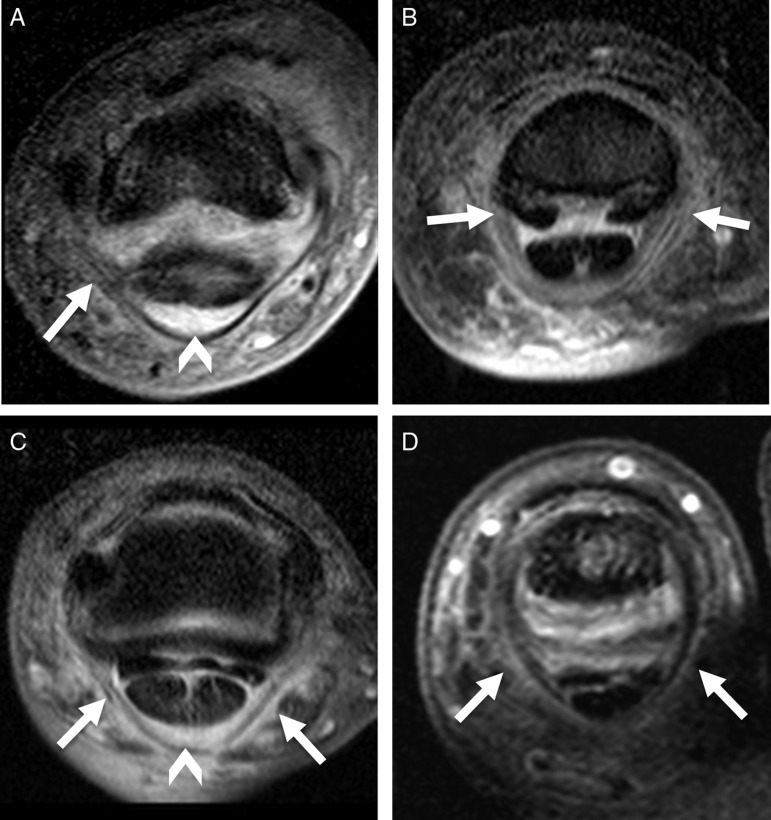
Finger pulleys and toe fibrous sheath abnormalities. Fat-saturated contrast-enhanced T1-weighted axial images. (A) Interphalangeal joint of the left thumb of a 57-year-old man. The A2 pulley region adjacent to the bone was unilaterally oedematous and ill-defined (white arrow). Area of enhanced high signal representing thickened flexor tenosynovium is also noted (arrowhead). (B) Distal site of proximal interphalangeal (PIP) joint of the left index finger of a 62-year-old man. There is oedematous change at bilateral A4 pulley attachments (white arrows). (C) PIP joint of the left index finger of same patient as (B). Symmetrical rim-like enhancement is observed around the A3 pulley (white arrows). The flexor tenosynovium is thickened as noted by the area of high signal (arrowhead). (D) PIP joint of the right 2nd toe of same patient as (2C) showing oedematous change of the fibrous sheath (white arrows).

Flexor tenosynovitis was seen in 3 fingers and 6 toes of 9 patients with dactylitis (75%). Of those, 7 patients showed mild to moderate diffuse tenosynovitis, and 2 patients mild focal tenosynovitis (figure 1C). In 3 fingers and 6 toes of 9 patients (75%), the finger pulleys or toe fibrous sheaths could be seen as low signal intensity structures wrapped around the flexor tendons. Two fingers and 4 toes (50% of all cases) showed signal intensity abnormalities in the pulleys including A2 (ill-defined with asymmetrical changes), A3 and A4 (oedematous with symmetrical changes) pulleys and the fibrous sheaths at 4 PIP joints (2 oedematous and 2 ill-defined) (figure 3). None of the normal controls had any signal changes in the tendon pulleys or fibrous sheaths, with only one having mild changes resembling flexor tenosynovitis (10%, p=0.004).

## Discussion

This study used hrMRI to explore the microanatomic basis for dactylitis in PsA patients. Herein, we demonstrate enthesitis is common in PsA dactylitis and that enthesopathy related to the flexor tendon pulleys and fibrous sheaths offers a novel explanation for the association with flexor tenosynovitis. Like Olivieri *et* al,^4^ ^5^ we saw no enthesitis at the flexor tendon insertions. However, we noted that the small entheses associated with the tendon pulleys and sheaths could be sites of enthesitis relevant to the tenosynovitis pathology. It is unclear if this observation is specific to dactylitis in PsA, as it had been previously noted that there was also absence of flexor tendon insertion enthesitis in osteoarthritis of the finger joints, although the tendon pulleys and sheaths were not examined.^14^ Two patients had no evidence of enthesitis at the collateral ligaments or extensor and flexor tendons, but these 2 patients had received corticosteroid therapy a few weeks before the scan due to clinical indications. Apart from this, we were unable to explain the differences between the dactylitic joints that demonstrated enthesitis and those that did not, likely due to the small sample size. However, in comparison, almost all the healthy control joints had no enthesitis. Although impossible to prove in man, our findings are supported by previous animal models of dactylitic-like swelling, where early changes were evident at insertions prior to the disease spreading to adjacent tissues.^15^ ^16^

Extensor tendonitis was identified adjacent to the phalangeal bone protuberance with inflammatory changes within the tendon at the point where it exerts pressure on adjacent bone during joint movement. This region has been dubbed as a ‘functional’ enthesis unit, where the friction of the extensor tendon enthesis against the bone appears to be related to inflammation.^11^ An epicentre of inflammation within this structure, rather than primarily within the synovium, offers a novel explanation for some of the extracapsular inflammatory change evident in dactylitis. It has been proposed that PsA is due to microtrauma, or ‘deep Koebner’ phenomenon, as recognised in skin psoriasis,^17^ an observation that has been supported by animal model data.^18^ Although this study did not indicate a predominance of abnormality at any one pulley due to the small number of joints scanned, the A2 pulley is a common site for injury in rock climbers, suggesting that this area is subjected to a high friction load.^19^ Indeed the MRI changes in injury in rock climbers bear some remarkable similarities with the changes reported in the present study.^20^

The limitation of this study was the small numbers of subjects. Nevertheless, the high-resolution nature of the MRI allowed adequate depiction of changes in the digits required for the study.

In conclusion, we have demonstrated inflammatory changes at digital pulleys and tendons, suggesting a form of ‘functional’ enthesitis that helps explain the nature of enthesitis in dactylitis. This conforms to the knowledge that enthesitis is characteristic of PsA and explains that diffuse swelling along the digits observed in dactylitis is linked to polyenthesitis.
